# Smoking and primary total hip or knee replacement due to osteoarthritis in 54,288 elderly men and women

**DOI:** 10.1186/1471-2474-14-262

**Published:** 2013-09-05

**Authors:** George Mnatzaganian, Philip Ryan, Christopher M Reid, David C Davidson, Janet E Hiller

**Affiliations:** 1 Faculty of Health Sciences, Australian Catholic University, Room 8.70, Level 8, 250 Victoria Parade, East Melbourne, Victoria, VIC, 3065, Australia; 2 Discipline of Public Health, School of Population Health, The University of Adelaide, Adelaide, South Australia, Australia; 3 Data Management & Analysis Centre, The University of Adelaide, Adelaide, South Australia, Australia; 4 Centre of Cardiovascular Research & Education in Therapeutics, Monash University, Melbourne, Australia; 5 Royal Adelaide Hospital, Emeritus consultant orthopaedic surgeon, Adelaide, South Australia, Australia

**Keywords:** Total joint replacement, Smoking, Socioeconomic status, Exposure misclassification, Sensitivity analysis

## Abstract

**Background:**

The reported association of smoking with risk of undergoing a total joint replacement (TJR) due to osteoarthritis (OA) is not consistent. We evaluated the independent association between smoking and primary TJR in a large cohort.

**Methods:**

The electronic records of 54,288 men and women, who were initially recruited for the Second Australian National Blood Pressure study, were linked to the Australian Orthopaedic Association National Joint Replacement Registry to detect total hip replacement (THR) or total knee replacement (TKR) due to osteoarthritis. Competing risk regressions that accounted for the competing risk of death estimated the subhazard ratios for TJR. One-way and probabilistic sensitivity analyses were undertaken to represent uncertainty in the classification of smoking exposure and socioeconomic disadvantage scores.

**Results:**

An independent inverse association was found between smoking and risk of THR and TKR observed in both men and women. Compared to non-smokers, male and female smokers were respectively 40% and 30% less likely to undergo a TJR. This significant association persisted after controlling for age, co-morbidities, body mass index (BMI), physical exercise, and socioeconomic disadvantage. The overweight and obese were significantly more likely to undergo TJR compared to those with normal weight. A dose–response relationship between BMI and TJR was observed (P < 0.001). Socioeconomic status was not independently associated with risk of either THR or TKR.

**Conclusion:**

The strengths of the inverse association between smoking and TJR, the temporal relationship of the association, together with the consistency in the findings warrant further investigation about the role of smoking in the pathogenesis of osteoarthritis causing TJR.

## Background

The incidence of total hip replacement (THR) and total knee replacement (TKR) has steadily increased over the past two decades and continues to rise as global populations grow [1-3]. In both men and women the procedure rates increase with age as patients reach their late 70s, after which the rates decline [3]. Lower limb total joint replacement (TJR) has become an effective and successful treatment for osteoarthritis (OA) of the hip and knee which is the most common musculoskeletal disorder to cause pain and disability in elderly populations and is the leading cause of this procedure [4]. Besides old age, some of the independent risk factors for this disorder include female gender [1,3], obesity [5], physical activity [6], and never-smoking [7-9]. However, the reported association of smoking with increased or decreased risk of osteoarthritis or total joint replacement has not been consistent [7-12]. Smoking has variously shown a negative association with OA [7,8] or TJR [9], a positive association with OA [10] or TJR [11], and no significant association with OA [12]. It has been suggested that the inverse association between smoking and TJR due to OA may be explained by various confounding factors such as body weight. Obesity is a major risk factor for OA [5,7] or TJR [9] and because body mass index often decreases with increasing duration of smoking [13,14], smokers who, in general, may be leaner than the non-smokers may be less likely to develop OA. Other proposed confounders of the inverse association are physical activity [6,15] and socioeconomic status (SES) [16]. The association of socioeconomic disadvantage with lower rates of joint replacements has been reported. People coming from such disadvantaged backgrounds often smoke more and are more likely to suffer from tobacco-related co-morbidities [17]. Similarly, compared with more affluent population groups, such patients may wait longer for surgery and may also have less access to TJR procedures [16,18]. The inverse association is further explained by probable misclassification bias of the smoking status, confounding by unknown factors, and by selection biases of the controls as suggested by Hui et al. [19].

In a previous analysis, we found an independent inverse dose–response relationship between duration of smoking and risk of undergoing a total joint replacement in 11,388 elderly men coming from the population-based cohort - the Health In Men Study (HIMS) [9]. This inverse association persisted after adjusting for confounding factors including age, co-morbidities, body weight, physical exercise, and various socioeconomic and demographic factors and also after accounting for the competing risk of death. One limitation of our previous study was that the data included only men and therefore the results were not generalizable to women. Furthermore, that study did not account for possible misclassification biases. To examine this association in another independent sample and evaluate if it existed also in women, we used a much larger sample of 54,288 elderly men and women belonging to the Second Australian National Blood Pressure Study (ANBP2) [20]. We hypothesized that if a possible association were to be supported between smoking and lower risk of either THR or TKR, such an association needed to be observed again in men but also in women in the independent ANBP2 sample. In this analysis, we further ran one-way and probabilistic sensitivity analyses (PSA) [21,22] to account for potential biases related to uncertainties in the classification of 1) the smoking status, and 2) the socioeconomic disadvantage scores.

## Methods

### Ethics statement

Ethical approval for the study was obtained from the Human Research Ethics Committees of 1) The University of Adelaide, 2) ANBP2 study, and 3) the Australian Institute of Health and Welfare (AIHW) National Death Index prior to commencement of study. All analyses used de-identified data. The need for informed consent was waived by the ethical committees due to de-identified data being used.

### Study population

The study population is drawn from the Second Australian National Blood Pressure study that was conducted at 1594 family medical practices throughout Australia [20]. The objective of the original study was to assess in hypertensive subjects 65–84 years of age, whether there was any difference in cardiovascular events over a 5-year treatment period between antihypertensive treatment with an ACE inhibitor-based regimen and treatment with a diuretic-based regimen. The general practitioners, who were approached and were willing to participate in the study, provided a list of potential eligible hypertensive subjects 65 years of age or older. During 1995–1998, a total of 54,288 subjects were screened for eligibility to participate in ANBP2.

### Study independent variables

At baseline screening of the original ANBP2 study, a questionnaire was completed that included socio-demographic data, presence of co-morbidities and lifestyle factors including current tobacco use, daily alcohol consumption, and engagement in physical activity. Height and weight and other measures of obesity (i.e., arm, waist and hip circumferences) were measured by a research nurse and body mass index (BMI) was calculated. The systolic and diastolic blood pressures were also measured. Both smokers and non-smokers were defined at baseline according to smoking status (yes/no) and all participants were followed for the study outcome from the same index date. General practitioners and research nurses also provided information on study participants’ co-morbidities. Participant-reported physical exercise variable was defined as engaging in weekly exercise that lasted more than 30 minutes. The socioeconomic status (SES) was measured by the Socioeconomic Index For Areas (SEIFA) which is based on data from the 1996 census for residential postcodes [23]. SEIFA is a composite index that ranks geographic areas across Australia in terms of their relative socio-economic advantage and disadvantage based on census data, where lower scores indicate more disadvantaged areas and higher scores indicate more advantaged areas. The score, which has been validated by the Australian Bureau of Statistics [23], is constructed using a number of different variables that indicate both advantage (i.e. high income, having a degree qualification) and disadvantage (i.e. unemployment status, low income, not enough bedrooms). For example, an area could have a low score if there are, among other things, many households with low incomes, or many people in unskilled occupations. This index is frequently used in Australian epidemiological studies where individual measures of socioeconomic status are not available.

### TJR and mortality ascertainment

The electronic records of the initially screened 54,288 men and women were linked to the Australian Orthopaedic Association National Joint Replacement Registry (AOA NJRR) [24] to detect primary total hip or knee replacements due to osteoarthritis from 1 September 1999 till 31 December 2010. In this study we considered TJR as a surrogate indicator of severe OA. Follow-up for TJR did not commence at baseline screening (1995–1998) since at baseline joint replacement procedures were still not registered in a national registry. Mean time from baseline screening to national complete capture of lower limb joint replacements by the AOA NJRR was 2.3 (SD 0.6) years [median of 2.2 years].

All-cause mortality was ascertained through linkage with the Australian Institute of Health and Welfare (AIHW) National Death Index [25].

### Statistical analysis

**Figure 1:**
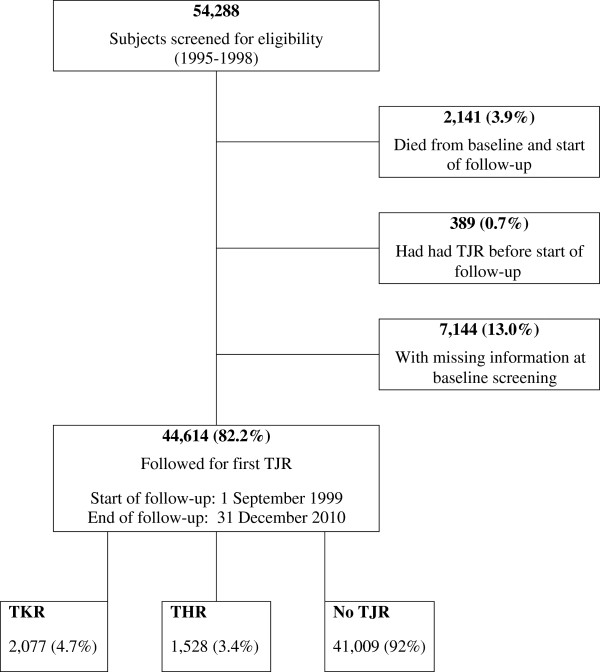
Flow chart of screening and follow-up for first total joint replacement due to osteoarthritis: the ANBP2 study.

We excluded from the analysis 1) participants who died between baseline screening and start of follow-up for TJR, 2) participants who reported having a TJR before start of follow-up, and 3) those who had missing baseline information. The remaining eligible attendants were followed until they experienced their first hip or knee replacement procedure due to osteoarthritis or died or were right censored at the end of follow-up (December, 2010) (Figure 1). Only the first lower limb replacement procedure was considered.

Total joint replacement was separately modelled for males and females on age, Charlson Co-morbidity Index, body mass index, socioeconomic disadvantage, smoking, physical exercise, alcohol intake, screening systolic and diastolic blood pressures and presence of a mental co-morbidity at baseline using competing risk regressions (CRR) as defined by Fine and Gray [26]. The analyses assessed the effect of predictors on the hazard of the subdistribution for TJR (the "subhazard") while accounting for the competing risk of death since the study population was elderly and death represented a competing risk that reduced the number of individuals at risk of the event of interest, TJR [26-28]. The regression model for competing risks of Fine and Gray estimates the ratios of the hazards of the subdistributions, a natural extension of Cox modelling for hazards in the non-competing risks situation. The hazard of the subdistribution [for TJR] can be interpreted as the probability of observing the event of interest in the next time interval while knowing that either the event did not happen until then or the competing event did happen [26].

The Charlson Co-morbidity Index [29] used to adjust for co-morbidity was based on all co-morbidities reported by the general practitioner, the research nurse and the study participants. The study outcomes were also modelled using the individual co-morbid conditions that form the Charlson index. The method used to build the score was similar to that reported by Chaudhry et al. [30]. The original Charlson weights were used to form the co-morbidity index [31]. We further separately modelled THR and TKR using similar methods as explained above. The proportional hazard assumptions were tested in each of the models using Schoenfeld residuals.

### One-way and probabilistic sensitivity analyses

#### Misclassification of smoking

At baseline screening, the study participants were asked to state their current smoking status, a yes or no question. Misclassification of this variable could occur if, for example, a subject declines to reveal his/her smoking status, thus introducing uncertainty in the measure of sensitivity of this exposure variable. Sensitivity is defined as the probability of a true smoker being classified as a smoker. Another uncertainty relates to the specificity of the smoking variable. Specificity is defined as the probability of a non-smoker being classified as a non-smoker. Here, uncertainty may theoretically occur due to a coding error, or when subjects falsely claim to smoke. The latter is less likely because, generally, non-smokers do not usually claim to smoke, therefore the main uncertainty in the smoking exposure relates to the sensitivity measure.

Under the assumption that non-smokers are less likely to claim smoking, we conducted one-way sensitivity analyses to calculate the expected risk ratios of having a TJR associated with smoking under various sensitivity measures (i.e., 0.99, 0.95, 0.90, 0.80, 0.75, 0.70, 0.60 and 0.50), while holding the specificity constant. For example, a sensitivity of 0.90 would indicate a misclassification of 10%, i.e., 10% of the smokers declined to reveal their smoking status and were misclassified as non-smokers while in fact they did smoke.

Since uncertainty can theoretically affect the sensitivity as well as the specificity, as suggested by Jurek et al. [21] and Orsini et al. [22], the possible scenarios of different sensitivity and specificity values (for example 1.0 or 0.9 or 0.8 or 0.75) were assessed simultaneously using probabilistic sensitivity analysis (PSA) through Monte Carlo simulations with 20,000 replications. A misclassification-bias-adjusted relative risk was estimated under a variety of possible fixed sensitivities and specificities of smoking classification among those with and without TJR.

The one-way and probabilistic sensitivity analyses were undertaken under the assumption that misclassification of smoking status was not associated with having a total joint replacement.

#### Misclassification of socioeconomic status (SES)

As stated above, the SES was measured by the Socioeconomic Index For Areas (SEIFA) disadvantage score that was based on the residential postcode. Misclassification of SEIFA may occur due to different reasons such as coding error or wrong residential address. To account for this possible uncertainly, as explained above, we conducted a PSA.

All analyses were performed using Stata statistical program (version 11, Stata-Corp.).

## Results

### Exclusions

At baseline (1995–98), a total of 54,288 hypertensive men and women (mean age 72.9 (SD 5.1) years) were screened for eligibility (Figure 1). Over 70% of the subjects were screened during 1997–1998. For this current analysis, of all screened the following were excluded from the study: 1) 2,141 (3.9%) participants who died between baseline screening and start of follow-up for TJR, 2) 389 (0.7%) who reported having their TJR before start of follow-up, and 3) 7,144 (13.0%) participants with missing baseline information. The proportion of TJR procedures in participants with missing information was similar to those without missing information (8.4% versus 8.1% respectively, P = 0.4). Mean age of these two groups was similar, and female proportions were not different (54.3% versus 55.5% respectively, P = 0.1).

### Study population characteristics

Mean age at baseline was 72.8 (SD 5.0) years with a median age of 72 years ranging from 57 to 91 years. Of the 44,614 study participants, 3,535 (8%) reported smoking at baseline screening. Compared to all others, the current smokers were significantly younger, leaner, more likely to be males, less likely to exercise, and more likely to have higher Charlson co-morbidity indices (Table 1). Similarly, smoking prevalence was higher among disadvantaged socioeconomic groups showing a gradient of increasing proportions of smokers with decreasing SEIFA scores observed in all participants and also when stratified by obesity (defined as BMI > =30 kg/m^2^) (Table 2). No significant differences were observed in mean systolic or diastolic blood pressures between current smokers and non-smokers.

**Table 1:** Characteristics of study participants by smoking status defined at baseline screening

**Characteristic**			**Current smoking status**		**P value**
			**Yes smoking**	**Not smoking**	
			**N = 3,535 (7.9%)**	**N = 41,079 (92.1%)**	
Age, mean (SD)			71.4 (4.5)	73.0 (5.0)	<0.001
Male gender, %			52.6	43.8	<0.001
BMI, %		Kg/m^2^			
	Underweight	<18.5	3.3	1.1	
	Normal weight	18.5 – 24.9	44.5	35.2	
	Overweight	25 – 29.9	38.1	44.7	
	Obese	30 – 34.9	11.9	15.4	
	Morbidly obese	35 or more	2.3	3.6	<0.001
SES, %	1^st^ quintile – Lowest SES		27.3	19.7	
	2^nd^ quintile		19.7	19.8	
	3^rd^ quintile		19.6	20.1	
	4^th^ quintile		17.9	20.2	
	5^th^ quintile – Highest SES		15.6	20.2	<0.001
Charlson co-morbidity index, mean (SD)			0.4 (0.8)	0.3 (0.7)	<0.001
Physical exercise > 30 minutes per week, %			49.7	55.8	<0.001
Daily alcoholic drink, %			15.4	7.1	<0.001
Screening systolic blood pressure, mean (SD)			143.0 (19.4)	143.5 (18.7)	0.135
Screening diastolic blood pressure, mean (SD)			79.5 (10.3)	79.2 (9.9)	0.140

Abbreviations: *BMI* (body mass index), *SES* (socioeconomic status based on the distribution of Socio-Economic Indexes for Areas (SEIFA) in the cohort).

**Table 2:** Proportion of current smokers and TJR by BMI and socioeconomic status

	**1**^**st **^**quintile (Lowest SES)**	**2**^**nd **^**quintile**	**3**^**rd **^**quintile**	**4**^**th **^**quintile**	**5**^**th **^**quintile (Highest SES)**
	**N = 9,046**	**N = 8,848**	**N = 8,953**	**N = 8,911**	**N = 8,856**
BMI < 30 (N = 36,305)					
Current smokers, %	11.5	8.3^!!^	8.3^!!^	7.4^!!^	6.5^!!^
TJR, %	6.7	6.6	7.7^!^	6.8	7.4
BMI > =30 (N = 8,309)					
Current smokers, %	7.6	6.2	5.4^!^	5.3^!^	4.9^!^
TJR, %	11.2	14.2^!^	12.0	13.0	13.1
All (N = 44,614)					
Current smokers, %	10.7	7.9^!!^	7.7^!!^	7.1^!!^	6.2^!!^
TJR,%	7.7	8.1	8.5^!^	7.9	8.3

Abbreviations: *BMI* (body mass index), *SES* (socioeconomic status based on the distribution of Socio-Economic Indices for Areas (SEIFA) in the cohort), *TJR* (total joint replacement).

! 0.001 < P value < 0.05; !! P value < 0.001, comparison with Lowest SES (1^st^ SEIFA quintile).

### Total joint replacement

All study participants were followed for a mean of 8.6 (SD 3.4) years till experiencing first primary THR or TKR or death or censoring at the end of follow-up. Of the 44,614 participants, 1,528 (3.4%) had a total hip replacement and 2,077 (4.7%) had a total knee replacement (Figure 1). Those who had either a THR or TKR were significantly younger, healthier with lower Charlson co-morbidity indices, heavier with higher body mass indices, and were more likely to be female than those who did not undergo this procedure. Compared with non-smokers at baseline, current smokers were significantly less likely to undergo a TJR procedure observed in all SES groups, age groups, and also seen in the obese and non-obese (Table 3). The inverse association was more prominently seen among the obese participants.

**Table 3:** Proportions of TJR by BMI, SES, median age categories, and current smoking status

**BMI**	**SES categories**	**Number of participants**	**Age**	**Age**	**All ages**
			**57–72 yrs**	**73 + yrs**	
BMI < 30	*Low SES*				
	Current smoker	1,208	5.5	4.8	5.2
	Non smoker	10,567	8.2^!^	5.4	6.8^!^
	*Middle SES*				
	Current smoker	999	4.9	4.1	4.6
	Non smoker	11,074	8.8^!^	6.5	7.6^!^
	*High SES*				
	Current smoker	829	5.8	3.2	4.8
	Non smoker	11,628	9.1^!^	5.6	7.3^!^
	*All SES categories*				
	Current smoker	3,036	5.4	4.1	4.9
	Non smoker	33,269	8.7^!!^	5.8^!^	7.2^!!^
BMI > =30	*Low SES*				
	Current smoker	216	5.6	5.6	5.6
	Non smoker	2,907	14.6^!^	10.1	12.8^!!^
	*Middle SES*				
	Current smoker	160	8.8	3.5	6.9
	Non smoker	2,634	17.1^!^	9.7	14.0^!^
	*High SES*				
	Current smoker	123	9.2	8.0	8.9
	Non smoker	2,269	14.3	9.5	12.3
	*All SES categories*				
	Current smoker	499	7.5	5.1	6.8
	Non smoker	7,810	15.4^!!^	9.8	13.0^!!^
ALL	Current smoker	3,535	5.7	4.2	5.2
	Non smoker	41,079	10.2^!!^	6.5^!^	8.3^!!^

Abbreviations: *BMI* (body mass index), *SES* (socioeconomic status based on the distribution of Socio-Economic Indices for Areas (SEIFA) in the cohort; *Low SES* 1^st^ tertile, *Middle SES* 2^nd^ tertile, *High SES* 3^rd^ tertile), *TJR* (total joint replacement).

! 0.001 < P value < 0.05; !! P value < 0.001, the non-smokers were compared with the smokers in all categories.

To assess the independent association of smoking with risk of undergoing a TJR, we modelled TJR for men and women separately while controlling for the factors listed in Table 4. In a model that accounted for the competing risk of death, smoking at baseline was independently and inversely associated with lower risk of TJR in both men and women, although the association was stronger in men. Compared to all others, men and women who were smokers at baseline were respectively 40% and 30% less likely to undergo a TJR (adjusted-sHRs: 0.60, CI 95% 0.48-0.75 in men, and 0.70, CI 95% 0.56-0.86 in women). Risk of TJR was significantly higher among the overweight and obese showing a dose–response effect across BMI categories (P < 0.001) in both genders. Adjusting for BMI as a continuous variable and using different measures of obesity (i.e. weight and height or arm, waist and hip circumferences) produced similar associations (results not shown). Engaging in weekly physical exercise was a risk factor in men but not in women (Table 4).

**Table 4:** **Subdistribution hazard ratios of undergoing total joint replacement in males and females: multivariable competing risk regressions**^**! **^**- accounting for the competing risk of death (N = 44,614)**

		**Male**	**Female**
		**N = 19,864**	**N = 24,750**
		**TJR: 1,405 (7.1%)**	**TJR: 2,200 (8.9%)**
		**Death prior to TJR: 46.8%**	**Death prior to TJR: 34.2%**
**Covariates**		**sHR (95% CI), P value**	**sHR (95% CI), P value**
Age, continuous		0.96 (0.95–0.97), <0.001	0.96 (0.95–0.97), <0.001
Charlson Index, continuous		0.75 (0.68–0.84), <0.001	0.73 (0.66–0.81), <0.001
BMI categories	Kg/m^2^		
Normal weight	18.5-24.9 (ref)	1.00	1.00
Underweight	<18.5	0.41 (0.10–1.67), 0.214	0.31 (0.15–0.61), 0.001
Overweight	25–29.9	1.47 (1.22–1.76), <0.001	1.60 (1.39–1.83), <0.001
Obese	30–34.9	2.05 (1.67–2.52), <0.001	2.31 (2.00–2.67), <0.001
Morbidly obese	≥ 35	2.92 (2.15–3.97), <0.001	2.55 (2.09–3.09), <0.001
Current smoker, yes		0.60 (0.48–0.75), <0.001	0.70 (0.56–0.86), 0.001
Exercise > 30 minutes /per week, yes		1.13 (1.01–1.26), 0.035	1.00 (0.92–1.01), 0.966
SEIFA score, continuous		1.00 (1.00–1.00), 0.049	1.00 (0.99–1.00), 0.213
Screening SBP, continuous		0.99 (0.99–0.99), 0.010	0.99 (0.99–0.99), <0.001

^!^Also controlled for alcohol intake, and screening diastolic blood pressure.

Abbreviations: *BMI* (body mass index), *SEIFA* (Socio-Economic Indexes for Areas), *SBP* (systolic blood pressure), *TJR* (total joint replacement), *sHR* (subdistribution hazard ratio).

To assess the association of smoking with different joints, we further modelled THR and TKR separately and found similar independent inverse associations between smoking and TJR (Table 5), although the association was stronger in TKR. Similarly, the association of BMI was stronger with TKR than with THR. Multivariable models for either THR or TKR were also conducted separately for males and females with similar findings (results not shown). The multivariable analyses were similarly run using all 17 co-morbid conditions that form the Charlson Index. The inverse association between smoking and TJR persisted also when individual targeted co-morbid conditions were assessed. Since fitting a statistical model to the data as a function of many co-morbid conditions together with other study covariates may result in model over-fitting [32], we preferred to present the results with the single Charlson Index.

**Table 5:** **Subdistribution hazard ratios of undergoing total hip or knee replacement: multivariable competing risk regressions**^**! **^**- accounting for the competing risk of death (N = 44,614)**

		**Total hip replacement**	**Total knee replacement**
**Covariates**		**sHR (95% CI), P value**	**sHR (95% CI), P value**
Age, continuous		0.97 (0.96–0.98), <0.001	0.95 (0.94–0.96), <0.001
Female gender, yes		1.34 (1.21–1.49), <0.001	1.27 (1.16–1.39), <0.001
Charlson Index, continuous		0.79 (0.71–0.88), <0.001	0.70 (0.63–0.77), 0.001
BMI categories	Kg/m^2^		
Normal weight	18.5-24.9 (ref)	1.00	1.00
Underweight	<18.5	0.46 (0.22–0.97), 0.042	0.19 (0.06–0.58), 0.004
Overweight	25–29.9	1.46 (1.25–1.71), <0.001	1.68 (1.44–1.95), <0.001
Obese	30–34.9	1.66 (1.38–1.99), <0.001	2.81 (2.39–3.30), <0.001
Morbidly obese	≥ 35	1.57 (1.17–2.09), 0.002	3.72 (3.03–4.57), <0.001
Current smoker, yes		0.72 (0.58–0.90), 0.004	0.59 (0.48–0.73), 0.001
SEIFA score, continuous		1.00 (0.99–1.00), 0.115	1.00 (0.99–1.00), 0.083
Screening SBP, continuous		0.99 (0.99–0.99), 0.011	0.99 (0.99–0.99), <0.001

^!^Also controlled for alcohol intake, physical exercise activity and screening diastolic blood pressure.

Abbreviations: *BMI* (body mass index), *SEIFA* (Socio-Economic Indexes for Areas), *SBP* (systolic blood pressure), *TJR* (total joint replacement), *sHR* (subdistribution hazard ratio).

Interaction was assessed between smoking with either BMI or physical exercise but no statistically significant interactions were detected.

### Sensitivity analysis

Table 6 shows the observed and expected risk ratios of having a TJR associated with smoking using one-way sensitivity analyses to account for uncertainty in the probability of a true smoker being classified as a smoker (i.e., sensitivity). With specificity held constant, the analysis was separately run using different sensitivity measures of 0.99, 0.95, 0.90, 0.80, 0.75, 0.70, 0.60 and 0.50. The inverse association between smoking and risk of TJR remained statistically significant under uncertainty levels ranging from 1% to 40%. (Table 6) The risk ratio was not statistically significant when the sensitivity was set to be 0.50.

**Table 6:** Observed and expected risk ratios of having a total joint replacement associated with smoking: one-way sensitivity analysis accounting for uncertainty in the classification of smoking exposure

	**Level of uncertainty**^**!**^	**Sensitivity**^**!!**^	**Risk ratio (95% CI)**	**P value**
Observed risk ratio	-	-	0.62 (0.54 – 0.72)	<0.001
Expected risk ratios based on one-way sensitivity analyses^!!!^	1%	.99	0.66 (0.58 – 0.75)	<0.001
	5%	.95	0.76 (0.68 – 0.84)	<0.001
	10%	.90	0.82 (0.75 – 0.90)	<0.001
	20%	.80	0.89 (0.82 – 0.95)	0.001
	25%	.75	0.90 (0.84 – 0.97)	0.004
	30%	.70	0.92 (0.86 – 0.98)	0.009
	40%	.60	0.93 (0.88 – 0.99)	0.032
	50%	.50	0.94 (0.89 – 1.01)	0.073

^!^Uncertainty in the classification of smoking. The values represent hypothetical percentages of study participants potentially declining to reveal that they indeed did smoke.

^!!^Sensitivity is defined as the probability of a true smoker being classified as a smoker.

^!!!^The one way sensitivity analysis evaluated the risk ratio under various sensitivity measures while holding the specificity fixed.

Running the probabilistic sensitivity analysis produced a misclassification-bias-adjusted risk ratio of 0.34, 95% CI 0.02 – 0.60.

Running simulations to account for possible uncertainty in the classification of the SEIFA score resulted in similar results as the study real data. No independent associations were observed between the disadvantage SEIFA score and risk of TJR.

## Discussion

In a large sample of elderly men and women, we have found that smoking is independently and inversely associated with undergoing a THR or TKR due to osteoarthritis in both men and women. This inverse association was observed after controlling for major confounders and also after accounting for possible misclassification biases.

A link between smoking and a protective mechanism against osteoarthritis has been previously reported. Two in-vitro studies have shown that this inverse relationship may be associated with glycosaminoglycans (GAG) synthesis [33], and with increased anabolic activity of the chondrocytes in joint cartilage [34]. Nicotine was reported to upregulate glycosaminoglycan and collagen synthesis in a dose–response manner among smokers, thus inhibiting the degeneration of joint structures [34]. Using magnetic resonance imaging, a third study found a positive dose–response between pack-years of smoking and tibial cartilage volume in healthy volunteers [35]. Our analysis is the largest cohort study to report inverse associations between smoking and risk of either THR or TKR due to osteoarthritis in both males and females. This reduced risk persisted after adjusting for major confounders and also after accounting for possible misclassification in the smoking exposure variable. Our sensitivity analyses showed that under a wide range of uncertainty levels in the misclassification of smoking, the significant inverse association between smoking and TJR persisted. Only when the sensitivity of exposure to smoking was as low as 0.50, the simulation produced a non-significant result. A sensitivity of 0.50 would increase the study’s observed number of smokers at baseline from 3,535 to 24,075 smokers; a seven-fold increase which seems very unlikely.

In a meta-analysis that assessed the association of smoking with osteoarthritis, Hui et al. [19] evaluated 48 cross-sectional, case–control and cohort studies and found an overall significant inverse association between smoking and risk of OA (OR: 0.87, 95% CI 0.80-0.94). Nonetheless, these authors concluded that such a negative association was most probably false resulting from a possible selection bias of controls. The authors reported that the negative association was predominantly demonstrated in the case–control studies and suggested that the results could have been biased by the inclusion of controls from hospital settings where patients, in general, were more likely to have higher exposure to smoking than the general population. We agree that many of the conditions for which patients are hospitalized may be associated with smoking and the selection of controls from such a population can bias results of case–control studies of tobacco-related diseases as reported by Morabia et al. [36]. However, unlike most of the studies assessed by Hui et al., our study samples (both ANBP2 and our previous HIMS study) were community-based, and such selection biases were unlikely. Moreover, out of the 48 studies included in the Hui et al. meta-analysis, 40 (83%) were cross-sectional or case–control, and such study designs are less appropriate to investigate any temporal relationship between a certain exposure and outcome [37]. In contrast, our study is longitudinal and exposure to smoking preceded the event of interest.

Utilisation disparities of total joint replacement by various socioeconomic groups have been reported [16,18,38]. In addition to differences in co-morbidities and level of education, those belonging to a low SES status may be less willing to undergo this procedure compared with the more affluent. Similarly, disparities in undergoing a TJR procedure may be associated with socio-economic access factors, and expectations about the process and outcomes of the procedure [38,39]. Unlike these reports, our longitudinal study did not find significant differences in the utilisation rates of TJR by various socio-economic groups. Our study population was relatively old and, possibly, those coming from lower socio-economic groups were under-represented. This possible selective loss to follow-up may have resulted in biased estimates of socio-economic inequalities in the utilisation of TJR that may occur in a population with a wider age range [40]. Similarly, the SEIFA indices ranked socio-economic well-being of the populations within areas rather than individuals themselves. Any area can include both relatively advantaged and disadvantaged people. Using the postcode may have introduced some misclassifications; however, since the postcode was provided by the participants, any misclassifications were minimized which was also supported by the probabilistic sensitivity analysis.

### Strengths and limitations

This study has several strengths including its longitudinal follow-up design, its large sample of participants of both males and females, and the many years of past exposure to smoking in our elderly participants. The linkage of the participants' records to the national mortality index data allowed us to account for all deaths in our study population. Moreover, death which may be more common among the elderly and especially among the smokers was accounted for as a competing risk. To our knowledge, this study is the first to assess risk of the study outcome while accounting for uncertainty in the exposure to smoking.

However, the study has limitations. Our retrospective cohort study, which is the highest possible level of evidence to investigate the relationship between smoking and long-term outcomes, is not a randomised controlled trial, thus confounding from other unaccounted factors is always possible. Available data did not permit us to control for duration or intensity of smoking, nor for past history of traumatic injury or past stressful physical work. Information on the physical activity of the participants was self-reported and not validated. The relatively advanced age of the participants at baseline enabled us to account for the co-morbidities and also various measures of obesity present at baseline. Nonetheless, we could not account for change in weight or co-morbidities that may have occurred over the mean 8.6 (SD 3.4) years of follow-up. Notwithstanding, age, which is often considered the simplest co-morbidity score [41], was accounted for over the follow-up period. Our study considered TJR as a surrogate indicator of severe osteoarthritis (OA). We therefore excluded all those who had had a TJR procedure in the past. We could not exclude those who had had hip or knee OA at baseline. The complete national capture of all lower limb joint replacements by the AOA NJRR was 2.3 (SD 0.6) years (mean) after the recruitment of the ANBP2 participants. However, there is no evidence to indicate that the missed procedures were more likely to be among smokers. We used co-morbidities reported by the GPs, research nurses and study participants to calculate the Charlson Index. We had no access to medical charts and therefore these co-morbidities were not validated. If co-morbidity were underestimated, the risk of TJR among non-smokers could have been overestimated (given that the current smokers had more co-morbidities than the non-smokers). Nonetheless, we constructed this co-morbidity score (that is based on 17 co-morbid conditions including various chronic pulmonary and other diseases) using a similar approach as demonstrated by Chaudhry et al. [30] who found high agreement levels between reported co-morbid conditions and those recorded in administrative datasets. Another explanation is the possibility of selection biases prior to surgery. Heavy smokers may have a lower chance of being put forward for surgery because of medical concerns regarding worse outcomes in such patients. However, a survey that sought to find indications for THR or TKR as perceived by orthopaedic surgeons showed that the decision against surgery was mainly affected by patient age, co-morbidity, obesity, alcohol use, technical difficulties and lack of motivation among the patients. Smoking was not indicated as a factor that would sway the decision against TKR or THR [42]. Finally, the study population was relatively old and our findings may not be generalizable to younger patient populations.

## Conclusion

The causal relationship of smoking with increased morbidity and mortality from many illnesses is well established; our results imply no support for the habit. In our previous population-based study [9], we found an independent inverse association between duration of smoking and risk of undergoing a lower limb joint replacement in men, showing a dose–response relationship. In this current longitudinal epidemiological study, we wanted to validate our previous results on an independent sample and to investigate whether these associations persisted after accounting for misclassification biases. Using a large sample of 44,614 individuals, we found that smoking was independently and inversely associated with having a total hip or knee replacements in both men and women. This is the first epidemiological study to show such consistent results in 1) males and females, 2) older and relatively younger participants, 3) low, middle and high socioeconomic status groups, 4) obese and non-obese participants, 5) and in both total knee and total hip replacements. Similarly, this is the first study to demonstrate that the inverse association between smoking and risk of TJR was more prominently observed among the obese. The strengths of the inverse association between smoking and TJR, the temporal relationship of the association, together with the consistency in the findings warrant further investigation about the role of smoking in the pathogenesis of osteoarthritis leading to lower limb joint replacement.

## Abbreviations

AIHW: Australian Institute of health and welfare; AOA NJRR: Australian orthopaedic association National joint replacement registry; BMI: Body mass index; CRR: Competing risk regressions; GAG: Glycosaminoglycans; HIMS: Health in men study; OA: Osteoarthritis; PSA: Probabilistic sensitivity analysis; ANBP2: Second Australian National blood pressure study; SBP: Systolic blood pressure; SEIFA: Socioeconomic index for areas; SES: socioeconomic status; sHR: Subdistribution hazard ratio; THR: Total hip replacement; TJR: Total joint replacement; TKR: Total knee replacement.

## Competing interests

GM, PR, CMR, DCD, and JEH declare that they have no competing interests.

## Authors’ contributors

Conception and design (GM, PR, JEH, DCD); Analysis and interpretation (GM, PR), Drafting of the article and critical revision of the article for important intellectual content (GM, PR, JEH, DCD, CMR); Final approval of the article (GM, PR, JEH, DCD, CMR); Collection and assembly of data (PR, CMR). GM serves as the guarantor for the manuscript. All authors read and approved the final manuscript.

## Pre-publication history

The pre-publication history for this paper can be accessed here:

http://www.biomedcentral.com/1471-2474/14/262/prepub
